# Influence of Pediveliger Larvae Stocking Density on Settlement Efficiency and Seed Production in Captivity of *Mytilus galloprovincialis* in Amsa Bay, Tetouan

**DOI:** 10.3390/ani14020239

**Published:** 2024-01-12

**Authors:** Hafsa Janah, Adil Aghzar, Pablo Presa, Yassine Ouagajjou

**Affiliations:** 1 Research Team of Agriculture and Aquaculture Engineering (G2A), Polydisciplinary Faculty of Larache, Abdelmalek Essaadi University, Tetouan 93000, Morocco; hafsa.janah@etu.uae.ac.ma (H.J.); a.aghzar@uae.ac.ma (A.A.); 2 Amsa Shellfish Research Station, National Institute of Fisheries Research, Tetouan 93000, Morocco; 3 Laboratory of Marine Genetic Resources (ReXenMar), CIM—Universidade de Vigo, 36310 Vigo, Spain; pressa@uvigo.es

**Keywords:** hatchery, spat, post-larvae settlement, seed production, stocking density, *Mytilus galloprovincialis*

## Abstract

**Simple Summary:**

The development of cost-effective protocols with optimized rearing systems and conditions is an essential requirement to assure the economic feasibility of mussel production in hatchery systems. The Mediterranean mussel *Mytilus galloprovincialis* plays a relevant role on the scale of bivalve aquaculture, whose expansion has deep economic and environmental consequences. The current study establishes the protocols to improve pediveliger settlement, post-larvae production and growth, through the optimization of the stocking density of larvae and post-larvae in hatchery systems. This standardized protocol for mussel rearing can enhance the production of quality seed to supply the fattening industry as well as for environmental restoration.

**Abstract:**

In mussel hatchery systems, the settlement process is a crucial element influencing seed yield. The current study assayed the influence of five densities of competent pediveliger larvae on settlement success and post-larvae production. We showed an inverse relationship between density and settlement efficiency, e.g., an attachment success of 99.4% at the lowest density (35 larvae/cm^2^) but only 9% at the highest density (210 larvae/cm^2^). However, post-larvae production was higher at intermediate larvae densities (70 larvae/cm^2^). The reimplementation of treatments upon post-larvae density after 6 weeks post settlement showed that the lowest-density groups bore both the highest post-larvae growth rate (22.24 ± 4.60 µm/day) and the largest head batch (48% of the size distribution), as compared to the higher-post-larvae-density groups. These results highlight the importance of optimizing both pediveliger larvae density and post-larvae density, to maximize high-quality seed yield in local hatcheries. Current rearing technologies would assure a timely commercial seed production to protect natural sea rocky beds in Alboran Sea coasts.

## 1. Introduction

In recent times, bivalve hatchery production has attracted high interest due to its potential to mitigate environmental hazards caused by human activities [1,2,3]. Also, the rapid human growth demands more shellfish supply [4], which has begun to exert pressure on the bivalve industry due to its limited availability [5,6]. Traditionally, mussel seed supply for aquaculture production was supported by the hand scrapping of natural seed from rocky shores [7]. However, the unsustainable exploitation of intertidal habitats has led to a serious decrease in natural populations and a reduction in ecosystem diversity [7,8]. Thanks to the advancement in bivalve husbandry, multiple opportunities exist nowadays for the development of reliable and certified mussel seed, to cope with both the industrial demands and the rehabilitation of overexploited habitats [5].

The Mediterranean mussel *Mytilus galloprovincialis* is native to the Mediterranean Sea [9,10,11,12,13] and is the most distributed of its genus worldwide [9]. Studies have suggested that this mussel expanded on the Atlantic coast of Morocco [14,15,16], where its commercial production has recently been launched [17]. For instance, several ongoing research initiatives are being developed in Morocco because of biological, environmental and commercial concerns to assess the viability and pertinence of the promotion of the mussel industry. From the genetic insight, the dispersal pattern and connectivity of its Atlantic metapopulation have been described [16,18], as well as its coexistence with the green mussel *Perna Perna* in northern Africa [19]. From an ecological perspective, the suitability of this mussel as a bio-monitor of environmental fluctuations has been shown [20,21,22,23]. Also, investigations of its growth performance in different culture systems suggest that the offshore mussel technology (longline systems) can assure higher yield and is deemed to a convenient solution for seed supply [17,24,25]. 

The reproductive cycle of this species has been studied on a world scale for a better understanding of its ecophysiology [26,27], biology [28,29] and genetics [30]. For instance, broodstock conditioning in hatchery systems is key to improve the quality and quantity of commercial stocks [31]. Those studies have revealed a high potential innovation in mussel aquaculture development [32], especially to produce juveniles and reduce pressure on natural populations [33]. It is well known that mussel larval rearing is directly influenced by temperature [34], feeding regimes [35,36,37], stocking density [38,39], bacterial loads [40] and CO_2_ levels [41], all of which improve or worsen the final larvae performance. Irreversible physiological processes in the pediveliger larvae of bivalves lead to the transition from planktonic to benthic life throughout metamorphosis and settlement [42]. Much research has been devoted to those latter processes because of their importance for the viability of populations. For instance, the exposure of *M. edulis* and *M. galloprovincialis* pediveliger larvae to microbial films resulted in the induction of metamorphosis and further settlement [43]. Those processes are also influenced by chemical cues such as in the silver-lip pearl oyster *Pinctada maxima* [44], by diet composition such as in *M. galloprovincialis* larvae [45,46] and by stocking density such as in clam juveniles and many other invertebrates [38,47]. Namely, larvae stocking density adds to the intra-specific competition for space and food in marine organisms [48,49] and, therefore, it is a key factor influencing settlement success, as shown in pediveliger larvae of the pearl oyster [50], as well as in the clam *Meretrix meretrix* [51]. 

Stocking density in *Mytilus galloprovincialis* has been shown to be a primary factor influencing not only the performance of post-larvae cultured in captivity but also that of mussel spat cultured in longline systems. For instance, increasing the stock density decreased the production efficiency and increased the fattening time to reach the commercial size in *M. galloprovincialis* cultured in the Black Sea [52]. A negative effect of a high post-larvae density on bivalve seed performance has also been reported in other species where the highest post-larvae growth rate was achieved under their lowest initial density [53]. In addition, pilot experiments on mussel broodstock from La Atunara (southwestern Spain) using static hatchery systems have shown that a medium larvae density (25 larvae/mL) assured good growth and high seed yield [54]. Given the multifactorial influence on larvae production and performance such as organic matter accumulation, it is also recommended to evaluate the influence of post-larvae density on the posterior mussel performance in flow-through systems [54]. Therefore, in this work, we assess how the stocking density of competent pediveliger larvae affects their settlement rate and post-settlement production. We also address the effect of post-larvae density on the growth rate and size distribution over a 6-week culturing experiment. This knowledge would help to optimize the large-scale production of mussel seed to cope with incipient industrial needs as well as for the restoration and enhancement of natural habitats in the Mediterranean region in conjunction with genetic background knowledge.

## 2. Materials and Methods

### 2.1. Spawning, Fertilization and Larval Rearing

*Mytilus galloprovincialis* broodstock originated from longline rearing systems in Amsa Bay, 35°31′59.5″ N 5°13′29.0″ W, Tetouan (Morocco). On 19 May 2022, fifty adult specimens from each sex were placed alternatively in hot (26 ± 1 °C) and cold (14 ± 1.5 °C) sea water baths for several cycles until they begun spawning and were placed in individual beakers with filtered and UV-treated seawater. Oocytes and spermatozoids were filtered separately through a 20 μm sieve, and oocyte quality (roundness) and spermatozoid motility were assessed under a binocular microscope. The density of gametes was assessed in a Malassez counting chamber (BR719005-1EA). The fertilization was performed in a 200 L polycarbonate tank using a ratio of 120 spermatozoids per oocyte. Total number of fertilized oocytes was defined as the average no. of fertilized oocytes from three sub-samples (25 µL) using a binocular microscope. Eggs were placed in a 1000 L tank containing filtered (0.2 µm) and UV-treated seawater at an initial density of 15 eggs/mL at 20 °C. Larvae feeding began from 48 hpf (hours post fertilization) to 17th dpf (days post fertilization). Food consisted of a microalgae mixture of increasing density with rearing age, i.e., 500–3000 cell/larvae of *Isochrysis galbana*, 500–1500 cell/larvae of *Chaetoceros calcitrans* and 500–1000 cell/larvae of *Tetraselmis suecica*.

### 2.2. Experimental Design

**Figure 1:**
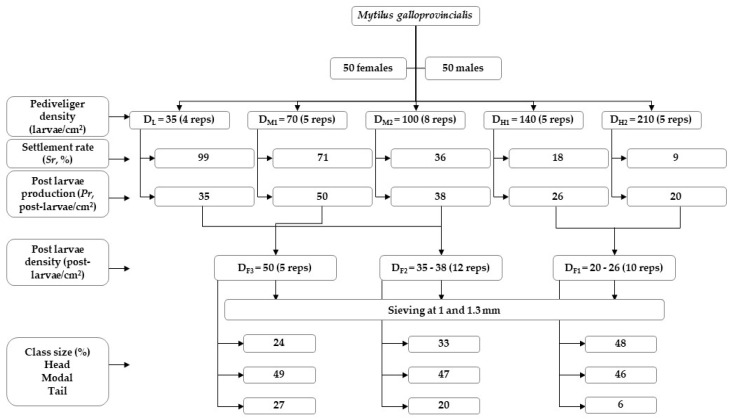
Experimental design and row results on larvae and post-larvae density testing in *Mytilus galloprovincialis*. D_L_, D_M1_, D_M2_, D_H1_, D_H2_, initial pediveliger stocking densities; reps, no. of replicates per density treatment; D_F1_, D_F2_, D_F3_, final post-larvae densities.

A panoramic view of the experimental design is given in Figure 1.

**Figure 2:**
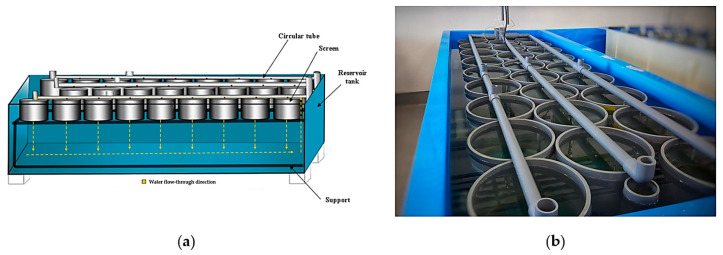
(**a**) Specific replicated structure implemented to monitor metamorphosis and settlement of mussel post-larvae; (**b**) detail of the downwelling system designed for rearing and tracking the larvae–post-larvae transition.

After the 17th dpf of larval rearing (pediveliger stage), larvae became competent for settlement and were distributed at a predesigned experimental density (Figure 2a). The settlement process was monitored in a series of cylinders (300 mm of diameter) bottomed with a 150 µm nylon mesh and placed into a 1200 L rectangular fiberglass tank. The rearing system (recirculating aquaculture system: RAS) provided filtered seawater, continuous aeration and controlled temperature (21.7 ± 0.69 °C) until the end of the experiment (Figure 2b). Post-larvae were fed a mixture of three strains with the total number of cells per milliliter varying progressively from 16,000 to 20,000 cell/mL of *I. galbana*, 4000 to 35,000 cell/mL of *C. calcitrans* and 4000 to 25,000 cell/mL of *T. suecica.* The physicochemical parameters recorded during the settlement process were pH (8.73 ± 0.08), salinity (36.75 ± 0.51 psu) and dissolved oxygen (7.08 ± 0.12 mg/L). Feeding, regular water renewal and cleaning of the culture system were performed every 48 h during the six weeks of the experiment (from 4 June to 16 July 2022). Settled post-larvae from each container were washed out, gently collected and counted using a profile projector (Nikon V-12B).

After 17 days of larvae rearing, the 1 m^3^ tank was sieved through a 150 µm mesh. The larvae were washed with microfiltered water and then resuspended in 1 L filtered-UV water. The no. of pediveliger was counted with a binocular on 3 samples of 25 µL each to obtain its concentration per mL. That density (*A*, No. of pediveliger/mL) was used to estimate the total no. of pediveliger (*Nx*) in the 1 L recuperation volume (*Vr*), as follows:

*Nx = A × Vr* (1)

About 4 million pediveliger larvae were produced and the corresponding aliquot was taken for each density treatment (*Dx*). Several larvae stocking densities were enforced in five treatments with replicates (rep), i.e., low density: D_L_ = 35 larvae/cm^2^ (4 rep), medium density: D_M1_ = 70 larvae/cm^2^ (5 rep) and D_M2_ = 100 larvae/cm^2^ (8 rep); and high density: D_H1_ = 140 larvae/cm^2^ (5 rep) and D_H2_ = 210 larvae/cm^2^ (5 rep). 

Each density treatment was defined using the following formula:

*Dx = Nx/S* (2)

where *Dx* is the density treatment, *Nx* is the number of pediveliger larvae needed to reach density *x*, and *S* is the surface of cylinder bottom (700 cm^2^).

The volume distributed to reach the required density treatment was calculated as

*Vx* = (*N*1 × *V*1)/*Nx* (3)

where *Vx* is the required volume to reach the density *Dx*; *N*1 is the initial number of pediveliger larvae; *V*1 is the total volume of pediveliger larvae; *Nx* is the number of pediveliger larvae needed to reach *Dx.*

**Figure 3:**
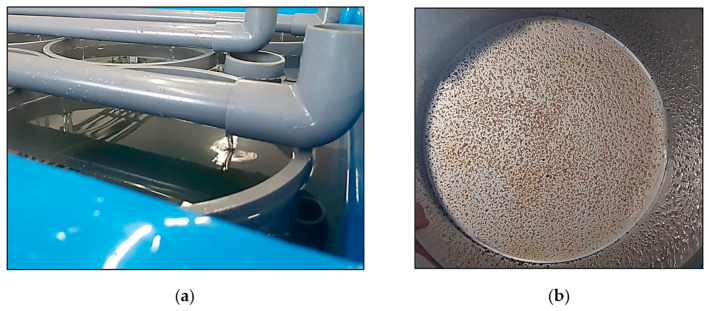
(**a**) Water flow through recirculating pipes into a nylon-mesh cylinder; (**b**) post-larvae settled in a screened nylon-mesh cylinder.

The settlement rate *(Sr)*, defined as the percentage of post-larvae settled on the nylon mesh cylinder (Figure 3a), was estimated on each replicate using the following formula:

*Sr (%)* = *(Nf/Ni)* × 100 (4)

where *Sr* is the settlement rate; *Nf* is the final number of produced post-larvae; *Ni* is the initial number of seeded larvae.

At the end of the 6-week experiment, the settled post-larvae from each nylon-mesh cylinder (Figure 3b) were collected and weighted and the final number of post-larvae *(Nf)* was calculated using the following formula:

*Nf = (Wt/Wi)* (5)

where *Nf* is the final number of produced post-larvae; *Wt* is the total weight of settled post-larvae per replicate; *Wi* is the individual average weight per post-larvae as estimated using a random subsample and the following expression:

*Wi = (Wsub/Nsub)* (6)

where *Wsub* is the total weight of the subsample per replicate; *Nsub* is the total number of post-larvae per subsample and replicate. The post-larvae production per cm^2^ was calculated as

*Pr* (post-larvae/cm^2^) *= (Nf/S)* (7)

where *Pr* is the post-larvae production (post-larvae/cm^2^); *Nf* is the final number of produced post-larvae; *S* is the surface area of cylinder mesh (cm^2^).

To assess growth in shell length, 45 pediveliger larvae and 75 post-larvae were measured per treatment using a profile projector (Nikon V-12B). The growth rate was calculated using the following formula:

*Gr* = (*M*_1_ − *M*_0_)/*t* (8)

where *Gr* is the growth rate; *M*_0_ is the initial average length of pediveliger larvae; *M*_1_ is the final average length of post-larvae; *t* is the number of days between the beginning and end of the experiment (42 days).

The five post-larvae production densities *(Pr*) obtained, i.e., 35, 50, 38, 26 and 20 post-larvae/cm^2^, were regrouped in three major density groups: low (D_F1_ = 20–26 post-larvae/cm^2^, 10 containers, five from each D_H1_ and D_H1_), moderate (D_F2_ = 35–38 post-larvae/cm^2^, 12 containers, four from D_L_ and eight from D_M2_) and high (D_F3_ = 50 post-larvae/cm^2^, 5 containers from D_M1_). All post-larvae within those three density groups were length-classified using two sieves of different meshes (1.0 mm and 1.3 mm) to produce a head batch >1.3 mm, a modal batch = 1.0–1.3 mm and a tail batch < 1 mm. The growth rate was estimated on 75 randomly sampled individuals from each batch.

### 2.3. Statistical Analyses

One-way ANOVA analyses (Fisher test, *p ≤* 0.05) as corrected with the Welch test [55] were applied to determine the effect of stocking density on post-larvae performance, settlement rate and percentage of size classes (the head, modal and tail batches). Boxplots were built to compare settlement rate, post-larvae production and size classes from each post-larval density. All the statistical analyses were conducted using R software, version 2021.05.29, Rcmdr package, version 2.7.2 and RStudio version 2022.12.0+353 [56].

## 3. Results 

### 3.1. Effect of Density on Post-Larvae Settlement (Sr)

**Figure 4:**
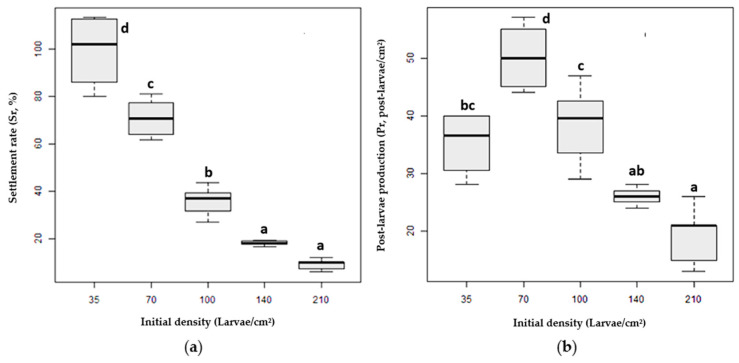
(**a**) Settlement rate (*Sr*, %); (**b**) post-larvae production (*Pr*, post-larvae/cm^2^) at different larvae stocking densities: D_L_ = 35 larvae/cm^2^; D_M1_ = 70 larvae/cm^2^; D_M2_ = 100 larvae/cm^2^; D_H1_ = 140 larvae/cm^2^; and D_H2_ = 210 larvae/cm^2^. A significant difference exists between treatments with different superscripts (a–d).

The settlement rate decreased with larvae density in a negative exponential trend (*r*^2^ = 0.98; *y* = 1.6452 × 10^−0.014*x*^) (Figure 4a). The highest settlement (99.4%) was observed at the lowest larvae density (D_L_ = 35 larvae/cm^2^), whereas the lowest settlement (9%) was observed at the highest density (D_H2_ = 210 larvae/cm^2^).

The ANOVA showed that the initial larval density significantly influenced larvae settlement (*F* = 93.45; df_num = 4; df_den = 8.9; *p* < 0.001) (Table 1). The settlement rate differed significantly between treatments except between the two highest ones, D_H1_ and D_H2_. 

### 3.2. Effect of Density on Post-Larvae Production (Pr)

Density D_M1_ exhibited the highest post-larvae production (*Pr*, D_M1_ = 50 post-larvae/cm^2^), whereas D_H1_ and D_H2_ showed the lowest productions, i.e., 26 and 20 post-larvae/cm^2^, respectively (Figure 4b). 

The initial larval density significantly influenced post-larvae production (*F* = 25.73; df_num = 4; df_den = 9.08; *p* < 0.001) (Table 2). Among the densities, a major difference in post-larvae production was observed between D_M1_ and the rest of the densities, whereas no difference was detected between (D_L_–D_M2_) and (D_H1_–D_H2_).

### 3.3. Effect of Stocking Density on Post-Larvae Shell Length Classes 

**Figure 5:**
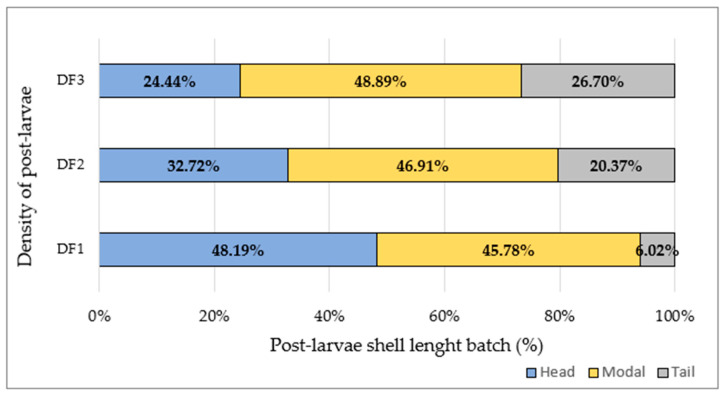
Percentage of post-larvae shell length batches (head, blue; modal, yellow; tail, grey) from three final post-larvae densities (D_F1_ = 20–26 post-larvae/cm^2^, D_F2_ = 35–38 post-larvae/cm^2^ and D_F3_ = 50 post-larvae/cm^2^).

Three post-larvae shell length batches were obtained from each post-larvae density distribution (D_F1_, D_F2_, D_F3_) (Figure 5). The lower the post-larvae density, the higher the share of the head batch, e.g., D_F1_, head batch = 48.19%. By contrast, the higher the post-larvae density, the higher the share of the tail batch, e.g., D_F3_, tail batch = 26.70%. 

### 3.4. Effect of Stocking Density on Post-Larvae Growth (Gr)

Significant differences in growth rate were observed between spat groups from different post-larvae densities (*F* = 16.683; df_num = 2; df_den = 250.97; *p* < 0.001). The highest growth rate (Gr) (22.24 ± 4.60 µm/day) was observed in the lowest-density treatment (D_F1_) (Table 3). 

One-factor ANOVA on post-larvae growth rate (µm/day ± SD) within the shell length batch (H, M, T) was significantly influenced by post-larvae density (D_F1_, D_F2_, D_F3_) in the head and tail batches (*F*= 31.66; df_num = 2; df_den = 89.42; *p* < 0.001 and *F* = 6.72; df_num = 2; df_den = 20.06; *p* < 0.01, respectively). No effect of post-larvae density was observed on the growth rate of the modal batch (*F* = 0.20; df_num = 2; df_den = 105.85; *p* = 0.83) (Table 4). The highest growth across treatments was observed in the head batches of the lowest post-larvae densities, i.e., D_F1_ and D_F2_ (Table 4).

## 4. Discussion

In mussels as in other bivalves, the transition from a planktonic to a sedentary life requires a sudden increase in food and space [57]. These needs make mussel nursery particularly challenging biologically as well as economically [33]. Several studies have been conducted to optimize early life cycle phases in both experimental pilot assays and commercial nursery systems [58,59,60,61,62]. The success of the settlement process is critical for seed production in quality and quantity and depends on multiple biological and physical factors. Particularly, stocking density is one of the key manageable factors able to enhance productivity [63]. The current study shows the correlation of pediveliger larval density on post-larvae settlement, production and growth in a hatchery RAS in the Alboran Sea coasts of northern Morocco (Amsa Bay).

The negative correlation observed between larvae stocking density and settlement rate suggests that seeding *M. galloprovincialis* culture substrates with more than ~35 larvae/cm^2^ (*Sr* = 99%) would not improve the settlement rate under local conditions. Moreover, increasing the density above 70 larvae/cm^2^ makes the settlement rate decay below 50%. Such a negative correlation between density and post-larvae settlement has been reported in numerous studies on bivalves. For instance, in the Asiatic hard clam *Meretrix meretrix*, more than 50% larvae were successfully settled [51], but settlement was delayed at higher densities (40–60 larvae/mL). The number of metamorphosed and settled larvae decreased at high stocking densities so that fewer larvae were able to release chemical cues to induce metamorphosis in the rest of the floating larvae [64]. The low post-larvae settlement under high larvae densities can result from space limitation as an essential element to optimize bivalve production in hatchery systems. For instance, space limitation in silver-lip pearl oyster juveniles (*Pinctada maxima)* provoked an aggregate settlement phenomenon which significantly reduced settlement rates [50]. In the zebra mussel (*Dreissena polymorpha*), it is believed that the aggregation phenomenon at high densities inhibits larvae site-selection as a natural behavior of mussel larvae prior to settlement [65]. Such behavior is time-limited, and consequently, unsettled larvae die soon afterwards [66].

The high settlement rates scored in the current RAS flow-through system (36–99.4%) are unexpectedly high as compared to previous observations, e.g., 6.3% settlement in Black-Lip Pearl Oyster (*Pinctada margaritifera*) [67], 50% in the clam Asiatic hard clam (*Meretrix meretrix*) [51] and >39% in the clam (*Venerupis pullastra*) in response to chemical induction [68]. It is worth noting that the flow generated in downwelling systems such as the current one, which provides velocity and turbulence, has been shown to be correlated to settlement success [69]. Previous studies were conducted on mussel settlement in response to different rearing factors. For instance, the highest settlement (49.3%) in the green mussel *Perna viridis* was achieved at a rearing temperature of 29 °C [70]. Also, velon screen was reported as the most suitable substratum for the settlement of green mussel *Perna viridis* post-larvae [71]. However, a hatchery study carried out on Australian blue mussels (*Mytilus* spp.) reported that optimal conditions for settlement may be achieved if other manipulations (food availability, airflow and type of substratum) are associated with stocking density [72].

Post-larvae production (*Pr*) has been reported as correlated to hatchery productivity in the Pacific oyster *(Crassostrea gigas)* [73]. In the present study, the significant relationship between larvae stocking density and post-larvae production indicates that tuning the former should not only consider settlement success but also yield. It is worth noting that the lowest pediveliger density (D_L_ = 35 larvae/cm^2^) which showed the best settlement rate (>99%) had a lower post-larvae production (*Pr* = 35 post-larvae/cm^2^) than the medium pediveliger density, i.e., D_M1_ = 70 larvae/cm^2^ and 50 post-larvae/cm^2^. Therefore, a stocking density that assures the highest larvae settlement does not assure the highest post-larvae production. Stocking at moderate densities between 70 and 100 larvae/cm^2^ is, in this case, the threshold beyond which enhanced post-larvae production was not attainable.

A negative correlation has been reported between density and size, growth rate and weight gain in cultured Cortez oyster (*Crassostrea corteziensis*) [48], in silver-lip pearl oyster juveniles (*Pinctada maxima*) [50] and in fishes, such as the dover sole (*Solea solea*) [74] or the seabream (*Sparus aurata*) [75]. It is worth noting that the same result appears masked in the current experiment, i.e., the positive correlation observed between the increase in initial pediveliger density (D_H1_ + D_H2_ = D_F1_) and the gain in shell length (Gr, µm/day) is counterintuitive. It indeed suggests that the higher the initial pediveliger density is, the lower both the settlement and production rate are, but the higher the growth rate of the surviving post-larvae becomes. The high growth rates achieved by post-larvae in the current study (e.g., D_F1_ = 20–26 post-larvae/cm^2^; *Gr* = 22.24 ± 4.60 µm/day) may also be caused by the downwelling system enforced. The flow velocity implemented can enhance the filtration activity of post-larvae and therefore their phytoplankton intake as previously reported in green-lipped mussel (*Perna canaliculus*) [61,76]. Despite the flow velocity also benefiting other density groups, the significantly slowed growth at high post-larvae density (D_F3_ = 50 post-larvae/cm^2^; *Gr* = 19.23 ± 3.75 µm/day) may be due to the digestive interference of metabolic waste, as shown in post-larval growth of the taquilla clam (*Mulinia edulis*) [77]. Nevertheless, this waste accumulation hypothesis is unlikely herein due to the water quality maintenance applied throughout this study, e.g., see the efficiency of recirculating aquaculture systems (RASs) to achieve optimized survival and biomass on the “tambaqui” *(Colossoma macropomum)* [78]. Therefore, we refer to overcrowding and subsequent aggregation as the potential factors slowing down mussel post-larvae growth as reported in the pearl oyster (*Pinctada fucata*) [79].

In this experiment, we have seen that post-larvae density is negatively correlated with growth rate. This relationship produced a growth variability between treatments but also a post-larvae growth heterogeneity between batches (head, modal, tail) within density treatments (D_F1_, D_F2_, D_F3_). It is worth noting that the lower the post-larvae density, the lower the inter-batch heterogeneity in shell length, e.g., D_F1_ = (48:46:6)% for the (head/modal/tail) batches, respectively. Heterogeneity is a byproduct of differential growth among individuals, which increases the size variance, and it is known as growth depensation [80]. This phenomenon is currently sorted out in aquaculture practice by size grading, implying higher labor costs and culture slots [81]. Therefore, the lower the heterogeneity in favor of shell length, the better the productivity would be in terms of reducing fattening time [24], e.g., the highest-density treatments (D_H1_ + D_H2_ = D_F1_) produced a 48% share of post-larvae reaching the fastest development time but only a 6% share of post-larvae with delayed growth. By contrast, the higher the heterogeneity within density treatment, the more dispersed was the shell length between size batches D_F2_ and D_F3_, requiring higher management costs and fattening time. As also reported in cupped oysters (*Crassostrea gigas*), a low stocking density assures a more homogeneous batch growth [82].

The usual mussel seed transfer to nursery systems around 1 mm in shell length increases the risk of seed mortality [83]. A head start with pediveliger larvae (average shell length of 260 µm), to spat with a shell length >1.3 mm in micro-nursery systems as obtained here in 6 weeks can produce an outstanding mussel culture performance. Specifically, the low post-larvae density treatment (D_F1_) resulted in a seed stock >1.4 mm in shell length average prior to their transfer to nursery systems when juveniles are stronger and more resilient to manipulation. 

## 5. Conclusions

The development of cost-effective protocols to produce high-quality mussel seed in hatchery systems requires the optimization of early rearing systems to assure viability. This work stresses the relevance of enforcing a pediveliger stocking density upon the production goal pursued. We show that an initial low density of larvae significantly enhanced the settlement rate (*Sr* = 70–90)% and post-larvae production (*Pr* = 35–50 post-larvae/cm^2^). However, high initial larvae densities (140–210 larvae/cm^2^) resulted in low *Sr* and *Pr*. Nevertheless, those low-density post-larvae cultures showed the highest growth rate among treatments (*Gr* = 22.24 ± 4.60 µm/day) and a size heterogeneity, i.e., 48% of post-larvae exhibiting the fastest development (head batch) and only 6% with a delayed growth (tail batch). The profitability of hatchery mussel production for enhancement and commercial production in the Alboran Sea of northern Morocco can be improved with the optimized stocking density protocols used in this study. Noteworthy, the resilience and consistency of our results are limited to the set of parameters established during this study (RAS, physicochemical environment, etc.). Therefore, future avenues related to the rearing system, hydrodynamics, type of settlement substratum, genetic improvement and probiotics are firm candidates to advance large-scale mussel production in captivity.

## Figures and Tables

**Table 1:** Results of ANOVA (one factor) for the settlement rate at different stocking densities (D_L_ = 35 larvae/cm^2^; D_M1_ = 70 larvae/cm^2^; D_M2_ = 100 larvae/cm^2^; D_H1_ = 140 larvae/cm^2^; and D_H2_ = 210 larvae/cm^2^). *Sr*, settlement rate; *F* = Fisher test; df_num = numerator degrees of freedom, df_den = denominator degrees of freedom, *** *p* < 0.001.

Initial Density (Larvae/cm^2^)	Mean *Sr* (±SD) ^1^	df_num	df_den	*F*	*p*-Value
D_L_	0.99 ± 0.16 ^d^	4.00	8.90	93.45	2.859 × 10^−7^ ***
D_M1_	0.71 ± 0.08 ^c^				
D_M2_	0.36 ± 0.05 ^b^				
D_H1_	0.18 ± 0.01 ^a^				
D_H2_	0.09 ± 0.02 ^a^				

^1^ A significant difference exists between treatments with different superscripts (a–d).

**Table 2:** ANOVA (one factor) for post-larvae production under different stocking densities (D_L_ = 35 larvae/cm^2^; D_M1_ = 70 larvae/cm^2^; D_M2_ = 100 larvae/cm^2^; D_H1_ = 140 larvae/cm^2^; and D_H2_ = 210 larvae/cm^2^), *F* = Fisher test; df_num = numerator degrees of freedom, df_den = denominator degrees of freedom, *** *p* < 0.001.

Initial Density (Larvae/cm^2^)	Post-Larvae Production (Mean *Pr* ± SD) ^1^	df_num	df_den	*F*	*p*-Value
D_L_	35.25 ± 5.85 ^b^	4	9.08	25.73	5.718 × 10^−5^ ***
D_M1_	50.20 ± 5.80 ^a^				
D_M2_	38.35 ± 5.93 ^b^ 26.00 ± 1.58 ^c^				
D_H1_	26.00 ± 1.58 ^c^				
D_H2_	19.20 ± 5.21 ^c^				

^1^ A significant difference exists between yields with different superscripts (a–c).

**Table 3:** Results of ANOVA (one factor) for post-larvae growth (shell length in µm/day) at different densities (D_F1_ = 20–26 post-larvae/cm^2^, D_F2_ = 35–38 post-larvae/cm^2^ and D_F3_ = 50 post-larvae/cm^2^). *F* = Fisher test; df_num = numerator degree of freedom, df_den = denominator degree of freedom *** *p* < 0.001.

Density (Post-Larvae/cm^2^)	Growth Rate (Mean *Gr* ± SD) ^1^	df_num	df_den	*F*	*p*-Value
D_F1_	22.24 ± 4.60 ^a^	2		16.68	1.576 × 10^−7^ ***
D_F2_	20.73 ± 4.65 ^b^		250.97		
D_F3_	19.23 ± 3.75 ^c^				

^1^ A significant difference exists between densities with different superscripts (a–c).

**Table 4:** Results of ANOVA (one factor) of the effect of post-larvae density on growth rate (Gr, µm/day) in the different batches (head, modal, tail). *F* = Fisher test; df_num = numerator degrees of freedom; df_den = denominator degrees of freedom; ^ns^ = not significant; ** *p* < 0.01, *** *p* < 0.001.

Batch	Density Group	Growth (*Gr* ± SD) ^1^	df_num	df_den	*F*	*p*-Value
Tail	D_F1_	12.76 ± 3.14 ^a^	2	20.06	6.72	2.25 × 10^−3^ **
	D_F2_	14.68 ± 0.84 ^b^				
	D_F3_	14.58 ± 1.14 ^b^				
Modal	D_F1_	19.27 ± 1.66 ^a^	2	105.85	0.20	0.82 ^ns^
	D_F2_	19.45 ± 1.86 ^a^				
	D_F3_	19.38 ± 2.08 ^a^				
Head	D_F1_	26.40 ± 2.55 ^b^	2	89.42	31.66	4.02 × 10^−11^ ***
	D_F2_	26.24 ± 2.19 ^b^				
	D_F3_	23.99 ± 0.97 ^a^				

^1^ Superscripts (a, b) indicate a significant difference between densities within batches.

## Data Availability

All data generated are contained either in the article or extendable upon request to the Corresponding Author.
